# STD Awareness Month — April 2014

**Published:** 2014-04-04

April is STD Awareness Month, an annual event calling attention to the impact of sexually transmitted diseases (STDs) in the United States. This month-long observance provides individuals, doctors, and community-based organizations the perfect opportunity to address ways to prevent some of nearly 20 million new cases of STDs that occur in the United States each year (1), costing the U.S. health-care system nearly $16 billion in direct medical costs (2) and placing a significant human and economic burden on the nation.

Although most sexually transmitted infections will not cause serious harm, some can lead to major health problems, such as infertility. Infection with a sexually transmitted pathogen can also make a person more susceptible to infection with the human immunodeficiency virus (HIV).

Behaviors such as not using condoms, having multiple sex partners, having anonymous sex partners, or having sex while under the influence of drugs or alcohol increase the risk for infection with a sexually transmitted pathogen. Lifestyle changes that reduce risk, regular STD screening, and prompt disease treatment are the most effective tools available to protect one’s health and prevent the spread of all STDs, including HIV.

During the month of April, CDC encourages clinicians to think about changes they might make to raise STD awareness among their patients and within their community. Learning resources for clinicians, patients, and community members about STDs are available from CDC at http://www.cdc.gov/std.
